# Components and Effectiveness of Adult Inpatient Psychiatric Rehabilitation Programs: A Scoping Review

**DOI:** 10.3390/healthcare13222971

**Published:** 2025-11-19

**Authors:** Panagiota Giannios, Fanie Chainey, Catherine Degré, Stéphanie Borduas Pagé, Alexandre Hudon

**Affiliations:** 1 Department of Psychiatry and Addictology, Faculty of Medicine, Université de Montréal, 2900 Bd Édouard-Montpetit, Montréal, QC H3T 1J4, Canada; panagiota.giannios@umontreal.ca (P.G.); stephanie.borduas-page.cemtl@ssss.gouv.qc.ca (S.B.P.); 2 Forensic Services, Department of Psychiatry, Institut Universitaire En santé Mentale de Montréal, 7401 Rue Hochelaga, Montréal, QC H1N 3M5, Canada; fanie.chainey.cemtl@ssss.gouv.qc.ca (F.C.); catherine.degre.cemtl@ssss.gouv.qc.ca (C.D.); 3 Department of Psychiatry, Institut Universitaire En Santé Mentale de Montréal, 7401 Rue Hochelaga, Montréal, QC H1N 3M5, Canada; 4 Centre de Recherche de l’Institut Universitaire En Santé Mentale de Montréal, 7331 Rue Hochelaga, Montréal, QC H1N 3V2, Canada; 5 Department of Psychiatry, Institut National de Psychiatrie Légale Philippe-Pinel, 10905 Boul Henri-Bourassa E, Montréal, QC H1C 1H1, Canada; 6 Groupe Interdisciplinaire de Recherche sur la Cognition et le Raisonnement Professionnel, Université de Montréal, 2900 Bd Édouard-Montpetit, Montréal, QC H3T 1J4, Canada

**Keywords:** mental health services, psychiatric rehabilitation, inpatients, mental disorders, program evaluation, recovery of function, adult psychiatry, treatment outcome

## Abstract

**Background/Objectives**: Inpatient psychiatric rehabilitation plays a critical role in the recovery of adults with serious mental illness (SMI), offering structured, supportive care to improve functioning and community integration. Despite its clinical relevance, there is considerable heterogeneity in how these programs are structured, delivered, and evaluated. This scoping review aimed to map the core components and reported outcomes of adult inpatient psychiatric rehabilitation programs and to identify elements associated with program effectiveness. **Methods**: A scoping review was conducted according to PRISMA-ScR guidelines. Four electronic databases (MEDLINE, Web of Science, PsycINFO, and Google Scholar) were systematically searched for articles published between inception and 2024. Studies were eligible if they described inpatient psychiatric rehabilitation programs for adults and reported on clinical, functional, or system-level outcomes. Data extraction included program characteristics, intervention components, effectiveness indicators, and study limitations. A qualitative thematic synthesis was conducted, and methodological quality was assessed using the Joanna Briggs Institute (JBI) critical appraisal tools. **Results**: Fifteen studies met the inclusion criteria. Core rehabilitation components included structured psychosocial interventions (e.g., CBT, psychoeducation), vocational and occupational support, recovery-oriented goal setting, and transitional care planning. Most programs demonstrated positive outcomes, including reduced readmissions, improved functional and psychosocial functioning, and enhanced quality of life. Effectiveness appeared to be influenced by the duration of rehabilitation, multidisciplinary team involvement, and continuity of care post-discharge. However, outcome measures varied considerably, and few studies used standardized assessment tools or included long-term follow-up. **Conclusions**: This review highlights common features of effective inpatient psychiatric rehabilitation programs and underscores the need for standardized outcome measures, implementation fidelity assessments, and longitudinal research. Findings can guide service planning, inform policy development, and support recovery-oriented inpatient care.

## 1. Introduction

Mental health disorders represent a growing global health concern, contributing substantially to the overall burden of disease and disability. The World Health Organization estimates that one in eight people worldwide lives with a mental health condition, with depression, anxiety, and schizophrenia ranking among the most disabling conditions [1]. When left untreated, these disorders often lead to chronic functional impairments, reduced life expectancy, social marginalization, and an increased risk of homelessness, substance use disorders, incarceration, and suicide [2,3]. Untreated mental illness also has substantial economic consequences, including lost productivity and increased healthcare utilization, costing the global economy nearly $1 trillion USD per year [4]. The long-term neglect of mental healthcare services has further exacerbated disparities, particularly for individuals with serious mental illness (SMI), who often remain excluded from employment, education, and meaningful social participation [5]. SMI is a term used to describe a group of psychiatric disorders that cause significant functional impairment and substantially interfere with one or more major life activities. It typically includes conditions such as schizophrenia spectrum disorders, bipolar disorder, and major depressive disorder with severe or persistent symptoms that require specialized, long-term treatment and support. Beyond diagnostic categories, SMI is characterized by chronicity, psychosocial disability, and an elevated risk of social exclusion, unemployment, and medical comorbidity. In the context of this review, the term SMI is used to encompass adults with enduring psychiatric disorders leading to significant impairment in social, occupational, or self-care functioning, consistent with definitions employed in previous rehabilitation research. These consequences underscore the urgent need for comprehensive, accessible, and person-centered interventions to support long-term recovery.

In response to these challenges, psychiatric rehabilitation programs have emerged as essential components of mental health service delivery. These programs are designed to support individuals with SMI in regaining the skills and confidence needed to live independently and participate fully in society [6]. Grounded in recovery-oriented care, psychiatric rehabilitation encompasses a diverse set of interventions, including (but not limited to) supported employment, housing support, social skills training, cognitive remediation, and peer-led services [7]. The goal of these programs is not merely symptom management, but rather to improve functioning, autonomy, and quality of life by aligning services with individuals’ personal recovery goals [8]. Rehabilitation services are typically multidisciplinary and community-based, facilitating social reintegration and reducing reliance on institutional care [9]. Increasingly, these approaches are considered indispensable to a modern mental health system that prioritizes personhood, dignity, and meaningful social inclusion.

Despite widespread endorsement of inpatient psychiatric rehabilitation, the literature reveals substantial heterogeneity in the design, implementation, and reported outcomes of these programs. There is currently no consensus on the essential components that constitute an effective rehabilitation model, nor are there standardized outcome metrics that capture recovery in a holistic, person-centered way [10,11]. Studies evaluating psychiatric rehabilitation vary in methodology, target populations, duration of intervention, and contextual factors, leading to inconsistent findings and limited generalizability [12]. Moreover, while some programs demonstrate positive effects on employment, housing stability, and quality of life, others report modest or no significant benefits, raising critical questions about what elements drive success [13,14,15,16]. This absence of uniformity affects the development of policy recommendations and limits the capacity of health systems to scale up best practices. A clearer understanding of the mechanisms and contextual features associated with effective inpatients psychiatric rehabilitation is therefore needed.

The objective of this scoping review is to map and synthesize the existing literature on the key components and elements of effectiveness of inpatients psychiatric rehabilitation programs for adults with serious mental illness. By identifying and categorizing the core intervention strategies, structural features, and outcome indicators associated with effective rehabilitation, this study aims to clarify the key elements of inpatient psychiatric rehabilitation programs and how their effectiveness are assessed. This synthesis will provide a baseline for developing evidence-informed guidelines and contribute to the optimization of rehabilitation services in mental healthcare systems. In doing so, it will also highlight critical gaps in the evidence base, guiding future research and service development toward more consistent, equitable, and recovery-oriented practices.

## 2. Materials and Methods

### 2.1. Search Strategies

A comprehensive scoping review was conducted to identify relevant studies on adult inpatient psychiatric rehabilitation published between inception and December 2024. The search encompassed multiple electronic databases, including MEDLINE (via PubMed), Web of Science and PsycINFO (via PsycNet). Studies identified from Google Scholar were also included in this review. This scoping review followed the PRISMA-ScR (Preferred Reporting Items for Systematic Reviews and Meta-Analyses extension for Scoping Reviews) guidelines to ensure methodological transparency and reproducibility. The search strategy incorporated a combination of free-text keywords and Medical Subject Headings (MeSH), focusing on core concepts such as psychiatric rehabilitation (e.g., “psychiatric rehabilitation,” “psychosocial rehabilitation,” “mental health recovery”), inpatient settings (e.g., “inpatient psychiatry,” “hospital-based care”), and serious mental illness (e.g., “schizophrenia,” “bipolar disorder,” “severe mental illness”). These terms were selected to reflect the primary aims of the review, namely to identify key components and effectiveness elements of psychiatric rehabilitation programs for adults in inpatient psychiatric settings.

Detailed search strategies for each database are provided in Supplementary Materials. The initial search strategy was developed by the lead author and refined in collaboration with a research librarian experienced in mental health systematic reviews. Searches were conducted by two reviewers (PG and AH) and results were cross-validated to ensure consistency and completeness. No language, geographic, or publication status restrictions were applied in order to capture a broad and inclusive range of evidence. A completed PRISMA-ScR checklist is available in Supplementary Materials [17]. This study was registered at: https://osf.io/7ntkh (accessed on 29 September 2025).

### 2.2. Study Eligibility

Studies were included based on the following criteria: (1) the population of interest consisted of adults (aged 18 and over) receiving care in an inpatient psychiatric rehabilitation unit or hospital-based rehabilitative setting; (2) the study described, evaluated, or examined components, models, or outcomes related to psychiatric rehabilitation; and (3) the primary focus of the intervention or program was related to recovery-oriented or functional rehabilitation in the context of SMI, such as schizophrenia, schizoaffective disorder, bipolar disorder, or other major psychiatric disorders. Studies were eligible regardless of methodological design (qualitative, quantitative, or mixed-methods) or outcome type (clinical, functional, psychosocial, quality of life, etc.). Only studies published in English or French were eligible for inclusion.

Studies were excluded if the population was limited to children or adolescents (under 18), if the setting was exclusively outpatient, community-based, or correctional without reference to inpatient rehabilitation services, or if the primary focus was on acute stabilization rather than rehabilitation. Studies that focused exclusively on substance use disorder treatment without addressing broader psychiatric rehabilitation goals were also excluded. Conference abstracts, editorials, commentaries, protocols without results, and non-peer-reviewed sources were not considered. Gray literature was excluded.

### 2.3. Data Extraction

Data extraction was conducted using a standardized form developed in Microsoft Excel, ensuring consistency and replicability in capturing relevant study characteristics. Two reviewers (PG and AH) independently extracted data from the included articles, and cross-verified all entries for consistency and accuracy. Any discrepancies in data interpretation or inclusion were discussed and resolved through consensus.

The following data items were systematically extracted from each study: (1) population sample characteristics (e.g., diagnosis, age range, sample size), (2) type of unit or setting (e.g., inpatient rehabilitation ward, long-term psychiatric hospital), (3) key components of the psychiatric rehabilitation program (e.g., psychosocial interventions, vocational support, skills training, peer involvement), (4) elements or indicators of program effectiveness (e.g., improvements in functioning, quality of life, hospital readmission rates), (5) limitations as identified by the study authors (e.g., small sample size, short follow-up duration, lack of control group), and (6) main outcomes and conclusions reported by the authors.

This structured approach facilitated thematic mapping and comparative synthesis of the included studies while maintaining methodological transparency.

### 2.4. Data Analysis

Data analysis was conducted using a structured and iterative approach consistent with thematic synthesis methods commonly applied in scoping reviews. Following data extraction, content from each study (particularly regarding the components of psychiatric rehabilitation and reported elements of effectiveness) was subjected to a process of inductive coding and thematic categorization. The quality of each study was assessed using the Joanna Briggs Institute (JBI) critical appraisal tools, based on their respective designs [18].

The variables charted included: population sample, type of unit, key components of psychiatric rehabilitation, elements of effectiveness, limitations, and main outcomes identified by the authors. These were reviewed to identify recurrent patterns, relationships, and meaning across studies. For example, recurrent features of psychiatric rehabilitation included biopsychosocial models, skills-based interventions, community care units, and person-centered planning. Elements of effectiveness were often framed in terms of improvements in quality of life, community reintegration, reduction in hospital readmission, or cost-effectiveness. These indicators were used to build a cross-study thematic map.

Thematic coding was carried out independently by two reviewers and then cross-validated. A Cohen’s kappa coefficient of 0.93 was calculated across core analytical fields, indicating a high level of interrater reliability. Minor discrepancies (especially in interpreting narrative outcome summaries) were resolved through consensus discussions to ensure consistency and fidelity to source material.

Codes were grouped into broader themes such as “Structural Components” (e.g., whole-system models, peer support, discharge planning), “Functional Outcomes” (e.g., housing stability, social integration, symptom management), and “Systemic Enablers/Barriers” (e.g., funding models, staff continuity, limitations due to retrospective designs or small samples). Patterns of convergence and divergence were examined across units (e.g., VA centers, CCUs, public hospitals), geographic contexts, and diagnostic populations.

A thematic synthesis matrix was developed to visually compare these findings across studies. Additionally, elements reported under Limitations and Main Outcomes were analyzed qualitatively to identify gaps in evidence, areas of inconsistency, and opportunities for program improvement. Through this process, the review generated a conceptual framework that maps core components of inpatient psychiatric rehabilitation and the multidimensional criteria by which their effectiveness has been evaluated.

## 3. Results

### 3.1. Description of Studies

**Figure 1:**
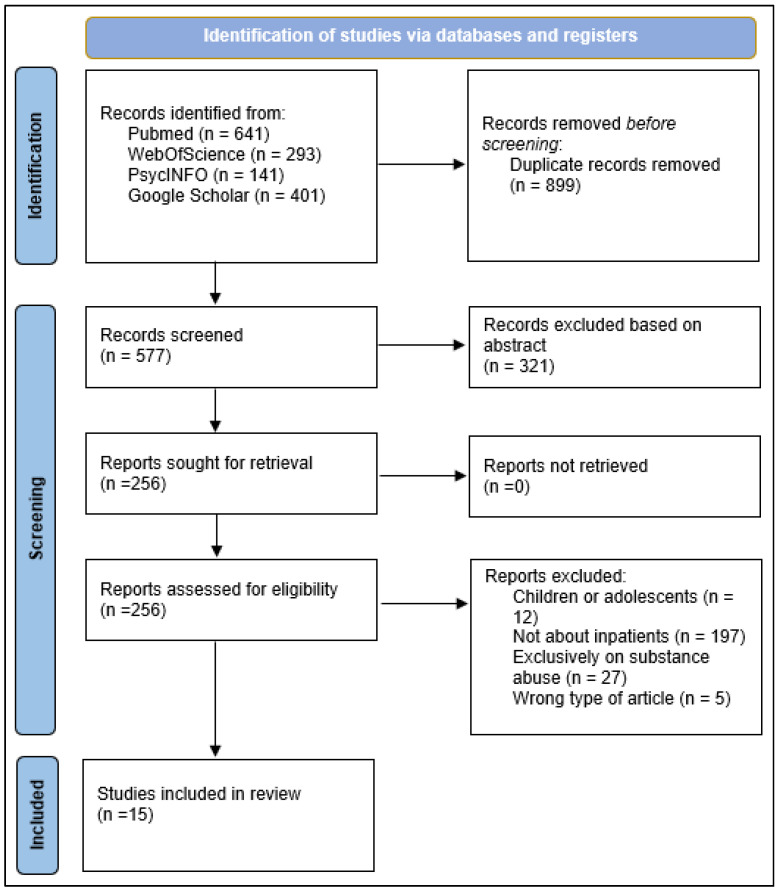
PRISMA Flowchart for the inclusion of studies.

The scoping review aimed to identify and synthesize studies examining the key components and effectiveness of psychiatric rehabilitation programs in adult inpatient settings. An initial literature search identified 1476 studies across the four databases and registers: PubMed (n = 641), Web of Science (n = 293), PsycINFO (n = 141), and Google Scholar (n = 401). After removing 899 duplicate entries, 577 unique studies were screened based on title and abstract. Of these, 321 were excluded for failing to meet preliminary relevance criteria. The remaining 256 reports were retrieved and assessed for eligibility in full-text form. Following full-text screening, 241 articles were excluded for the following reasons: study population restricted to children or adolescents (n = 12), absence of an inpatient context (n = 197), exclusive focus on substance use disorders (n = 27), or wrong publication type (n = 5). Overall, 15 studies met the inclusion criteria and were included in the final synthesis. A detailed overview of the screening and selection process is depicted in Figure 1. The specific details of the included studies are available in Supplementary Materials.

### 3.2. Study Characteristics and Populations

This scoping review included fifteen peer-reviewed studies conducted between 1990 and 2024 across a wide range of inpatient psychiatric rehabilitation settings. Most studies focused on adults diagnosed with severe mental illnesses such as schizophrenia, schizoaffective disorder, or bipolar disorder, often with complex needs including treatment resistance, comorbid substance use, cognitive impairments, or psychosocial instability. Settings ranged from veterans’ hospitals and general inpatient units to specialized community care units, forensic services, and long-stay institutions.

Sample sizes varied widely, from 22 patients in a retrospective evaluation by Bunyan et al. to over 500 in the national study led by Killaspy et al. [19,20]. Many populations shared characteristics of long-standing mental illness, multiple hospitalizations, and barriers to community reintegration.

### 3.3. Core Components of Psychiatric Rehabilitation

Despite marked heterogeneity in the design, setting, and intensity of inpatient psychiatric rehabilitation programs, a number of core therapeutic components consistently emerged across the reviewed studies. These components coalesced around five interrelated domains: structured psychosocial interventions, occupational and vocational support, individualized goal-setting, continuity of care and discharge planning, and a recovery-oriented, person-centered philosophy of care. Many programs explicitly aimed not just to reduce symptoms, but to restore functioning, enhance autonomy, and support meaningful reintegration into the community.

#### 3.3.1. Structured Psychosocial Interventions

Psychosocial therapies were a central feature in nearly all rehabilitation models. These included a combination of cognitive behavioral therapy (CBT), social skills training, psychoeducation, and group-based interventions focused on illness management and emotional regulation. For example, VanMeerten et al. implemented a robust package of evidence-based interventions within a U.S. Veterans Affairs setting, comprising family psychoeducation, social skills training, wellness management and recovery (WMR), and peer support programs as part of an integrated psychosocial rehabilitation service [21]. This multimodal model targeted cognitive, interpersonal, and emotional domains to improve both functional and community outcomes.

Similarly, Gonda et al. emphasized assertive psychosocial engagement techniques to address treatment-resistant schizophrenia [22]. Their inpatient approach included reality orientation training, activity scheduling, and structured feedback mechanisms to enhance insight, compliance, and therapeutic alliance. These were often paired with motivational interviewing to address ambivalence and enhance intrinsic motivation.

Several studies (such as those by Sim et al. and Pinkney et al.) also described the routine use of psychoeducation groups, particularly focusing on diagnosis, medication management, coping strategies, and relapse prevention [23,24]. Sim et al. further emphasized that implementing such interventions required frontline staff to be trained in consistent messaging, shared goals, and communication across disciplines [23].

#### 3.3.2. Occupational and Vocational Activities

Rehabilitation programs often incorporated occupational therapy and pre-vocational or vocational supports aimed at building daily routines, self-efficacy, and readiness for employment or volunteering. Awara et al. described a system-wide inpatient rehabilitation model that explicitly integrated daily living skills training and goal-oriented activity planning into care plans, which were collaboratively developed with patients and reviewed weekly [25,26].

Parker et al., in their large-scale evaluation of Community Care Units (CCUs) in Queensland, emphasized the inclusion of structured leisure and vocational activities (such as gardening, kitchen duties, and supported job placements) as part of their model of residential rehabilitation [27]. These activities were not simply adjunctive but were central to fostering independence and a sense of purpose, especially for patients with prolonged illness duration or institutional histories.

#### 3.3.3. Goal-Setting and Recovery Planning

Personalized goal setting, frequently operationalized through tools such as recovery plans, wellness action plans, or care pathways, was a recurring feature of effective rehabilitation. Killaspy et al., in their national evaluation, highlighted the use of structured recovery planning tools in inpatient and community rehabilitation units across England [20]. These plans were multidisciplinary in nature and involved the patient actively in identifying short- and long-term objectives in areas such as housing, relationships, symptom management, and meaningful activity.

In Bunyan et al.’s forensic rehabilitation cohort, the authors reported that collaborative goal-setting and progress monitoring played a critical role in reducing aggression, enhancing engagement, and lowering the risk profile of patients [19]. Similarly, Pinkney et al. described individualized support planning post-discharge as essential to sustaining community tenure and perceived quality of life [24].

#### 3.3.4. Continuity of Care and Discharge Planning

Effective programs embedded discharge planning from the outset and promoted continuity with community mental health teams. Awara et al. reported that early discharge planning, initiated within the first month of admission, was associated with improved community integration and fewer readmissions [25]. Their whole-system rehabilitation approach included coordinated case conferences, transitional housing referrals, and family meetings as routine practice.

Parker et al. similarly found that patients discharged from CCUs with coordinated aftercare and housing supports had significantly lower rates of hospital readmission. The integration of peer workers and community navigators to assist with this transition was an innovation described in several studies, particularly in the Sim et al. and VanMeerten et al. models [21,23].

#### 3.3.5. Recovery-Oriented, Person-Centered Philosophy

Most programs explicitly endorsed a recovery-oriented ethos, with care emphasizing patient autonomy, empowerment, and strength-based perspectives rather than merely symptom control. Killaspy et al. described rehabilitation services grounded in the QUIRC framework (Quality Indicator for Rehabilitative Care), which assesses domains such as therapeutic environment, recovery-based practice, and rights-based approaches [20].

Gonda et al. and Koval et al. both argued that success in rehabilitation was deeply linked to relational and environmental factors, such as trust in staff, consistency in care, and the physical and social milieu of the unit [22,28]. Programs that fostered shared decision-making, non-hierarchical relationships, and hope-oriented discourse were more likely to report improved functional outcomes and patient satisfaction.

Even within forensic and complex care contexts, such as those described by Bunyan et al. and Sim et al., staff training in trauma-informed care, motivational strategies, and flexible boundary-setting was critical to engaging patients with high needs or ambivalence toward treatment [19,23].

### 3.4. Elements of Effectiveness

The effectiveness of inpatient psychiatric rehabilitation programs was assessed using a diverse array of outcome indicators, reflecting the multidimensional nature of recovery. Most studies employed a combination of clinical, functional, and service utilization outcomes, while some also incorporated subjective and experiential measures such as patient satisfaction and perceived quality of life. Collectively, these outcomes revealed not only short-term clinical gains but also meaningful long-term improvements in autonomy, community integration, and reduced reliance on acute services.

#### 3.4.1. Reduction in Hospital Readmissions and Service Utilization

A commonly reported marker of effectiveness across studies was a reduction in hospital readmissions, length of stay, or emergency service utilization following discharge from inpatient rehabilitation. Parker et al. found that patients discharged from CCUs were significantly less likely to be readmitted within a one-year period, especially when care was coordinated with community mental health services [27]. Awara et al., in a Canadian context, reported similar findings, noting that patients who completed the rehabilitation program demonstrated extended community tenure and lower rates of psychiatric hospitalization compared to control groups [25].

Bunyan et al., working in a forensic inpatient context, showed a statistically significant decrease in bed days over the year following discharge, as well as a reduction in total service costs, largely attributed to fewer crisis episodes and acute admissions [19]. These findings suggest that rehabilitation not only improves outcomes but may offer substantial economic value by reducing system burden.

#### 3.4.2. Functional and Psychosocial Improvements

Beyond service-level metrics, many studies documented substantial gains in social functioning, independent living skills, and psychosocial well-being. VanMeerten et al. demonstrated that Veterans participating in structured psychosocial rehabilitation programs (including peer support, wellness recovery action planning, and vocational assistance) exhibited measurable improvements in functioning and community participation [21].

Gonda et al. reported increased treatment engagement, better compliance with therapeutic regimens, and enhanced insight into illness, all of which are critical mediators of long-term stability [22]. In the study by Sim et al., patients referred to inpatient rehabilitation showed improvements in self-regulation, social interaction, and goal-setting capacity, as captured through clinical documentation and staff ratings over time.

Several studies, such as those by Pinkney et al. and Koval et al., highlighted the importance of interpersonal and community-oriented gains, including the ability to maintain housing, re-establish familial or peer relationships, and develop meaningful routines [24,28].

#### 3.4.3. Patient-Centered and Subjective Outcomes

Many studies emphasized subjective indicators of recovery, such as patient satisfaction, perceived quality of life, hope, and empowerment. Pinkney et al., in their early Canadian study, found that individuals discharged from long-term psychiatric care and placed in community housing reported high levels of satisfaction with their autonomy and social opportunities, even when clinical symptoms persisted. These findings argue for the value of holistic metrics that encompass personal meaning and experiential domains of recovery [24].

Koval et al. similarly argued that success in psychiatric rehabilitation should be judged not solely by clinical remission, but by the ability of individuals to lead “self-determined, socially connected, and purposeful lives” [28]. This broader conceptualization of effectiveness was echoed in the study led by Killaspy et al., which used a comprehensive quality indicator framework to assess both objective and subjective domains of care quality [20].

#### 3.4.4. Duration of Treatment and Dose–Response Effect

Several studies observed that longer lengths of stay in rehabilitation were associated with greater post-discharge stability and reduced relapse. Bunyan et al. and Awara et al. both noted that patients with longer rehabilitation exposure (typically 6 to 12 months) demonstrated stronger functional gains and fewer readmissions, suggesting a dose–response relationship [19,25]. However, this finding was often balanced against concerns about institutional dependence and the need for timely discharge planning, as reported by Sim et al. and Parker et al. [23,27].

#### 3.4.5. Contextual Moderators and Implementation Factors

Effectiveness was also moderated by contextual factors such as program fidelity, staff training, and patient engagement strategies. Sim et al. demonstrated that when barriers to referral (e.g., unclear staff roles, patient ambivalence) were addressed through quality improvement interventions, rehabilitation uptake and outcomes improved markedly [23]. Likewise, VanMeerten et al. found that programs incorporating peer-led components and structured goal-setting had better patient retention and functional improvement metrics [21].

Conversely, studies that lacked robust aftercare or transitional support (such as those cited by Gonda et al.) frequently reported rebound hospitalization or disengagement from services, underscoring the importance of system continuity in determining long-term effectiveness [22,29].

### 3.5. Heterogeneity and Limitations

While the overall findings of this scoping review support the value of inpatient psychiatric rehabilitation, the heterogeneity of study designs, populations, and intervention models significantly complicates efforts to generalize conclusions or establish comparative effectiveness. Across the reviewed literature, key areas of variability included the inclusion criteria for participants, the structure and intensity of interventions, the metrics used to assess outcomes, and the length and rigor of follow-up periods.

#### 3.5.1. Variability in Patient Populations and Diagnoses

Studies differed markedly in the clinical profiles and sociodemographic characteristics of included participants. Some, like VanMeerten et al., focused exclusively on veterans with SMI, while others such as Parker et al. and Bunyan et al. included individuals with dual diagnoses, forensic involvement, or long-standing treatment resistance [19,21,27]. Gonda et al.’s cohort was composed of inpatients with chronic schizophrenia, yet the baseline severity and functional impairment varied greatly, which may have influenced engagement and response to intervention [22].

In addition, programs varied in the stage of illness they targeted. Some emphasizing early recovery or first-episode psychosis (e.g., Sim et al.), while others focused on patients with extensive hospitalization histories or high relapse risk [23,30]. This lack of homogeneity in participant characteristics limits the comparability of results and raises questions about for whom specific models are most effective.

#### 3.5.2. Inconsistencies in Intervention Models and Program Fidelity

There was considerable divergence in the content, duration, and delivery of rehabilitation interventions. Some programs, like those described by Awara et al., Tsoutsoulis et al. and Killaspy et al., implemented structured, multi-component packages with standardized protocols and multidisciplinary staffing [20,25,31]. Others, such as those in Bunyan et al. or Pinkney et al., were more descriptive in nature, offering general psychosocial support without clearly defined manuals or fidelity assessment [19,24].

Moreover, very few studies formally evaluated program fidelity or treatment adherence, and even fewer assessed the dose–response relationship between intervention intensity and outcomes. This absence of standardized implementation metrics makes it difficult to determine whether inconsistent outcomes were due to patient factors, contextual barriers, or variability in how programs were delivered.

#### 3.5.3. Methodological Limitations and Study Designs

Several studies employed retrospective or non-comparative designs, which limit the ability to infer causality or control for confounding variables. For instance, Gonda et al. relied on a descriptive cohort model without control groups or statistical adjustment, and Bunyan et al., while reporting promising reductions in inpatient bed use, included a relatively small sample (n = 22) and lacked subgroup analyses that might have clarified which participants benefitted most [19,22].

Even in larger studies like Killaspy et al.’s trial, the use of observational rather than experimental designs restricted causal inference [20]. Sim et al. applied a quality improvement framework, but acknowledged that their outcomes were based on internal clinical metrics rather than validated, blinded outcome measures [23]. As a result, interpretation is complicated by possible bias or subjective rating inflation.

#### 3.5.4. Outcome Measures and Follow-Up Durations

There was wide variability in the types of outcomes assessed, ranging from administrative data (e.g., readmission rates, bed days) to subjective reports (e.g., patient satisfaction, quality of life) and functional indicators (e.g., housing stability, employment). Some studies, like Pinkney et al., focused almost exclusively on subjective outcomes, while others emphasized system-level utilization data without attention to patient experience [24].

Furthermore, follow-up durations varied considerably, from as short as 30 days (Sim et al.) to over one year (Awara et al., Parker et al.) [23,25,27]. In several cases, follow-up was either not clearly specified or relied on post-discharge service contact as a proxy for stability. This inconsistency undermines efforts to compare sustainability of benefits or identify long-term effects.

#### 3.5.5. Contextual and System-Level Barriers

Several studies highlighted systemic challenges that constrained the scalability and transferability of successful rehabilitation models [32]. Sim et al. reported barriers to referral, including unclear staff roles, patient ambivalence, and lack of interdepartmental coordination, all of which limited access to rehabilitation beds [23]. Bunyan et al. emphasized the impact of resource limitations and policy variability in forensic settings, where structural factors often dictated the length and content of rehabilitation independently of clinical need [19].

Other studies, such as those by Parker et al. and VanMeerten et al., implicitly acknowledged the influence of local service cultures, staffing models, and funding mechanisms on program effectiveness [21,27,33]. These factors that are difficult to measure systematically but critically shape outcomes. Few studies attempted to quantify or control for these macro-level influences.

### 3.6. Quality Assessment of the Studies

While the overall quality was moderate to high for most studies, heterogeneity in reporting and methodological rigor was noted. Quality appraisal was guided by eight key domains of the JBI appraisal tool: clarity of objectives and inclusion criteria, appropriateness of study design, robustness of data collection and outcome measurement, handling of confounding factors, transparency in reporting, and alignment with the scoping review’s focus [18].

Cohort and Observational Studies (e.g., Parker et al., Awara et al., VanMeerten et al., Killaspy et al.) generally met core criteria related to clear inclusion criteria, defined exposures (e.g., rehabilitation interventions), and objective outcome measures such as readmissions and functional scores [20,21,25,27]. For example, Awara et al. used a large sample and systematic matching to evaluate outcomes, satisfying criteria for internal validity [25]. However, several studies lacked control groups and did not adjust for potential confounders, limiting causal inferences. Follow-up periods were often short or inconsistently defined, reducing the strength of longitudinal conclusions.

Quasi-experimental and Pre-Post Intervention Studies (e.g., Sim et al., Bunyan et al.) demonstrated strong relevance to the review question and high fidelity in intervention implementation, but often scored lower on aspects such as sample size justification, statistical control for confounding variables, and use of validated instruments [19,23]. For instance, Bunyan et al. employed a well-documented intervention in a forensic setting, but the small sample size (n = 22) and lack of statistical modeling limited generalizability.

Qualitative and Mixed-Methods Studies (e.g., Koval et al., Pinkney et al.) were assessed based on credibility, transferability, dependability, and confirmability [24,28]. Koval et al. provided rich narrative accounts and alignment between theoretical orientation and data interpretation, although few studies described saturation processes or triangulation. Ethical reporting was variable, with some papers omitting mention of ethics board approvals or participant consent procedures.

Across all designs, reporting of limitations was relatively transparent. Nearly all studies acknowledged structural or contextual barriers to implementation, and many highlighted potential sources of bias or measurement error. However, few studies incorporated standardized fidelity tools or blinded assessments, and none utilized randomized allocation.

Overall, 14 of the 15 studies were rated as moderate to high quality, meaning they met most JBI criteria relevant to their design. Three studies were considered methodologically limited, primarily due to small samples, descriptive-only analysis, or lack of follow-up data. Two studies (Killaspy et al. and Awara et al.) stood out for their comprehensiveness, multi-site sampling, and structured use of standardized tools [20,25]. Importantly, all included studies contributed valuable insights into real-world psychiatric rehabilitation, despite methodological constraints.

## 4. Discussion

This scoping review mapped the structural components and indicators of effectiveness across fifteen inpatient psychiatric rehabilitation programs targeting adults with SMI. Despite marked heterogeneity in design, most studies converged on five core domains: structured psychosocial interventions, occupational and vocational support, goal-oriented recovery planning, early discharge coordination, and a recovery-oriented philosophy of care. These programs demonstrated a range of beneficial outcomes, including reduced hospital readmissions, enhanced functional independence, improved quality of life, and increased patient satisfaction. Importantly, the effectiveness of these interventions was moderated by contextual features such as program fidelity, staffing, and community integration mechanisms. While the overall quality of the included studies was moderate to high, inconsistencies in methodology and outcome measures highlight the need for greater standardization in both the design and evaluation of inpatient rehabilitation services.

### 4.1. Comparison with Prior Works

These findings align with the body of literature that supports person-centered, recovery-focused mental health interventions as foundational to psychiatric rehabilitation. Leamy et al. identified five key recovery processes: connectedness, hope, identity, meaning, and empowerment [10]. The centrality of psychosocial engagement, vocational support, and collaborative care planning echoes the “CHIME” framework and emphasizes the need for rehabilitation services to extend beyond symptom control toward fostering social inclusion and self-determination [10]. Further, as shown in studies by Drake et al. and Bond et al., implementing structured, evidence-based practices (e.g., CBT, supported employment) in inpatient settings can yield sustainable improvements when properly adapted and integrated within multidisciplinary teams [34,35].

Beyond the scope of individual rehabilitation units, the literature suggests that broader system-level factors significantly shape the success of psychiatric rehabilitation. Harvey et al. stress the importance of coordinated community-based models that provide continuity of care post-discharge, including housing, employment support, and peer involvement [7]. The observed success of discharge planning and transitional support in this review is consistent with research by Munthe-Kaas et al., who found that integrated service models (including housing-first or assertive community treatment) dramatically reduce homelessness, rehospitalization, and crisis service use among people with SMI [13]. Likewise, Kreuter et al. argue that addressing social determinants, such as housing and income stability, within health service delivery can amplify the long-term impact of clinical interventions [14]. These findings highlight that inpatient rehabilitation must be conceived as part of a broader continuum of recovery infrastructure, not a siloed or terminal episode of care.

Emerging research also emphasizes the need for individualized outcome tracking in rehabilitation settings. Traditional metrics such as readmission rates and bed days (though useful) may not fully capture the experiential and functional recovery priorities of patients. Slade et al. and Sklar et al. advocate for integrating subjective indicators (e.g., hope, autonomy, perceived support) alongside objective metrics in evaluating rehabilitation effectiveness [16,36]. This recommendation is particularly relevant in light of the current review, where many included studies demonstrated substantial gains in domains like housing stability, treatment engagement, and social participation, even in the absence of full symptom remission. Incorporating validated person-reported outcome measures into routine assessment could enhance our understanding of what matters most to patients and better guide program development.

Beyond traditional psychosocial interventions, recent advances highlight the importance of social cognition and cognitive remediation in optimizing rehabilitation outcomes. Social cognition, encompassing emotion recognition, theory of mind, and attributional style, has emerged as a key determinant of interpersonal functioning and community reintegration among individuals with SMI. Structured interventions such as *Social Cognition and Interaction Training* specifically target these domains and have shown additive benefits when combined with cognitive remediation therapy, leading to improved social functioning, treatment engagement, and overall quality of life [37]. Integrating these approaches within inpatient psychiatric rehabilitation can therefore enhance recovery trajectories by addressing the cognitive-affective mechanisms that underlie relational and functional impairments.

As highlighted in Barlattani et al., disruptions in social cognition (exacerbated by stress, neuroinflammation, and social isolation during the COVID-19 pandemic) further underscore the necessity of embedding social cognitive assessment and remediation within modern rehabilitation models [37]. This neurocognitive perspective bridges psychosocial rehabilitation and affective neuroscience, offering a more comprehensive, biopsychosocial framework for recovery-oriented inpatient care.

### 4.2. Practical Recommendations

The findings from this scoping review offer valuable guidance for improving the design, implementation, and evaluation of inpatient psychiatric rehabilitation programs for adults with SMI. Despite heterogeneity in program models and outcome metrics, several consistent themes emerged across the literature that point to actionable strategies for optimizing recovery-oriented care. Translating these insights into practice requires attention not only to the clinical content of interventions but also to systemic, organizational, and relational dimensions that shape patient outcomes. The following practical recommendations are intended to support mental health professionals, administrators, and policymakers in enhancing the quality, effectiveness, and person-centeredness of psychiatric rehabilitation services.

#### 4.2.1. Integrate Structured Psychosocial Interventions as Core Program Elements

Structured interventions such as CBT, social skills training, and psychoeducation should be consistently embedded into inpatient rehabilitation programming. These interventions not only improve symptom management but also enhance social competence and self-efficacy. Clinicians should receive training and supervision to ensure fidelity to evidence-based models.

#### 4.2.2. Embed Recovery-Oriented, Person-Centered Practices

Rehabilitation should adopt a strength-based, recovery-oriented philosophy that emphasizes patient autonomy, goal setting, and empowerment. This requires staff to work collaboratively with patients to define meaningful goals and actively support identity reconstruction and hope-building as part of care planning. Embedding peer support workers and using personalized care plans can facilitate this shift.

#### 4.2.3. Enhance Transition Planning and Post-Discharge Linkages

One of the clearest predictors of effectiveness was continuity of care post-discharge. Inpatient teams should collaborate early with community resources (housing, vocational services, outpatient providers) to develop structured transition plans. Programs should consider implementing step-down models or community care units that allow gradual reintegration into less restrictive environments.

#### 4.2.4. Address Rehabilitation Duration and Intensity to Clinical Needs

Findings suggest a dose–response relationship: longer stays, when clinically indicated, are associated with more durable outcomes. Programs should move away from arbitrary time limits and instead use individualized readiness assessments to determine discharge timing. Functional markers, rather than just symptom reduction, should guide this process.

#### 4.2.5. Incorporate Multidisciplinary Teams with Defined Roles

Effective programs consistently involved multidisciplinary teams, including psychiatrists, nurses, occupational therapists, social workers, and peer specialists. Clarifying each team member’s role (particularly around psychosocial vs. medical interventions) enhances coherence and improves engagement. Regular interdisciplinary case reviews can support this structure.

#### 4.2.6. Monitor Outcomes Beyond Symptom Remission

Programs should move beyond clinical symptomatology as the primary indicator of success. Use of Patient-Reported Outcome Measures, including assessments of quality of life, autonomy, hope, and community participation, offers a more comprehensive view of recovery. Incorporating these into routine evaluations can guide care and support funding justification.

#### 4.2.7. Address System-Level Barriers and Resource Constraints

Finally, administrators and policymakers must recognize that program fidelity and effectiveness are contingent on adequate staffing, space, and time. Interventions to address referral biases, reduce turnover, and allow for meaningful therapeutic engagement are essential. Institutional commitment to rehabilitation (not merely containment) is necessary to operationalize best practices.

### 4.3. Limitations

Despite its contributions, this scoping review has several limitations. First, the inclusion of only English and French language articles may have excluded relevant international research, especially from low- and middle-income countries where innovative inpatient rehabilitation models may be underrepresented in indexed journals. Second, while the review included studies spanning different continents and health systems, the heterogeneity in study design, population, and outcome metrics limited direct comparability and precluded meta-analytic synthesis. Third, most included studies lacked long-term follow-up or control groups, and very few examined implementation fidelity, which constrains conclusions about causality or scalability. Additionally, gray literature and unpublished data were excluded, potentially omitting important program evaluations conducted outside academia. Future research should prioritize standardized outcome frameworks, implementation science approaches, and longitudinal designs to strengthen the evidence base for inpatient psychiatric rehabilitation.

## 5. Conclusions

Given the complexity and diversity of functional impairments observed among adults with serious mental illness, inpatient psychiatric rehabilitation programs have been developed to address a wide range of recovery needs. From the fifteen studies included in this scoping review, several core components were identified (such as structured psychosocial interventions, vocational support, recovery-oriented planning, and transitional care coordination), each contributing in varying degrees to clinical and functional improvement. These elements appear to enhance patient autonomy, reduce hospital readmissions, and foster more sustainable community integration. The findings highlight the importance of multidisciplinary, individualized, and recovery-focused approaches in promoting meaningful rehabilitation outcomes. Despite encouraging evidence, the current body of literature remains limited in consistency and scope. This review offers a foundational synthesis of inpatient rehabilitation models and their outcomes, which can inform practice and service design. Future research is warranted to evaluate program fidelity, long-term effectiveness, and the integration of person-centered outcomes across diverse care settings.

## Data Availability

No new data were created or analyzed in this study. Data sharing is not applicable to this article.

## Supplementary Materials

The following supporting information can be downloaded at https://www.mdpi.com/article/10.3390/healthcare13222971/s1: Table S1: Complete search strategies for each database; Table S2: PRISMA-Scr checklist.; Table S3: Details of the identified studies.

## Abbreviations

The following abbreviations are used in this manuscript:

SMI	Serious Mental Illness
CBT	Cognitive Behavioral Therapy
CCU	Community Care Unit
PRISMA-ScR	Preferred Reporting Items for Systematic Reviews and Meta-Analyses Extension for Scoping Reviews
JBI	Joanna Briggs Institute
WHO	World Health Organization
NHS	National Health Service
VA	Veterans Affairs

## References

[1] World Health Organization (2022). World Mental Health Report: Transforming Mental Health for All.

[2] Walker E.R., McGee R.E., Druss B.G. (2015). Mortality in mental disorders and global disease burden implications: A systematic review and meta-analysis. JAMA Psychiatry.

[3] Kessler R.C., Berglund P., Demler O., Jin R., Merikangas K.R., Walters E.E. (2005). Lifetime prevalence and age-of-onset distributions of DSM-IV disorders in the National Comorbidity Survey Replication. Arch. Gen. Psychiatry.

[4] Chisholm D., Sweeny K., Sheehan P., Rasmussen B., Smit F., Cuijpers P., Saxena S. (2016). Scaling-up treatment of depression and anxiety: A global return on investment analysis. Lancet Psychiatry.

[5] Thornicroft G., Brohan E., Rose D., Sartorius N., Leese M., INDIGO Study Group (2009). Global pattern of experienced and anticipated discrimination against people with schizophrenia: A cross-sectional survey. Lancet.

[6] Rössler W. (2006). Psychiatric rehabilitation today: An overview. World Psychiatry.

[7] Harvey C., Zirnsak T.-M., Brasier C., Ennals P., Fletcher J., Hamilton B., Killaspy H., McKenzie P., Kennedy H., Brophy L. (2023). Community-based models of care facilitating the recovery of people living with persistent and complex mental health needs: A systematic review and narrative synthesis. Front. Psychiatry.

[8] Drake R.E., Bond G.R., Essock S.M. (2009). Implementing evidence-based practices for people with schizophrenia. Schizophr. Bull..

[9] Dalton-Locke C., Marston L., McPherson P., Killaspy H. (2021). The Effectiveness of Mental Health Rehabilitation Services: A Systematic Review and Narrative Synthesis. Front. Psychiatry.

[10] Leamy M., Bird V., Le Boutillier C., Williams J., Slade M. (2011). Conceptual framework for personal recovery in mental health: Systematic review and narrative synthesis. Br. J. Psychiatry.

[11] Jaiswal A., Carmichael K., Gupta S., Siemens T., Crowley P., Carlsson A., Unsworth G., Landry T., Brown N. (2020). Essential Elements That Contribute to the Recovery of Persons With Severe Mental Illness: A Systematic Scoping Study. Front. Psychiatry.

[12] McQuaid J.R., Marx B.P., Rosen M.I., Bufka L.F., Tenhula W., Cook H., Keane T.M. (2012). Mental health assessment in rehabilitation research. J. Rehabil. Res. Dev..

[13] Munthe-Kaas H.M., Berg R.C., Blaasvær N. (2018). Effectiveness of interventions to reduce homelessness: A systematic review and meta-analysis. Campbell Syst. Rev..

[14] Kreuter M.W., Thompson T., McQueen A., Garg R. (2021). Addressing Social Needs in Health Care Settings: Evidence, Challenges, and Opportunities for Public Health. Annu. Rev. Public Health.

[15] Bond G.R., Drake R.E., Mueser K.T., Latimer E. (2001). Assertive community treatment for people with severe mental illness: Critical ingredients and impact on patients. Dis. Manag. Health Outcomes.

[16] Slade M. (2009). Personal Recovery and Mental Illness: A Guide for Mental Health Professionals.

[17] Tricco A.C., Lillie E., Zarin W., O’Brien K.K., Colquhoun H., Levac D., Moher D., Peters M.D.J., Horsley T., Weeks L. (2018). PRISMA Extension for Scoping Reviews (PRISMAScR): Checklist and Explanation. Ann. Intern. Med..

[18] Munn Z., Barker T.H., Moola S., Tufanaru C., Stern C., McArthur A., Stephenson M., Aromataris E. (2020). Methodological quality of case series studies: An introduction to the JBI critical appraisal tool. JBI Evid. Synth..

[19] Bunyan M., Ganeshalingam Y., Morgan E., Thompson-Boy D., Wigton R., Holloway F., Tracy D.K. (2016). In-patient rehabilitation: Clinical outcomes and cost implications. BJPsych Bull..

[20] Killaspy H., Dalton-Locke C., Clarke C.S., Leavey G., Igoumenou A., Arbuthnott M., Barrett K., Omar R. (2024). Assessing the clinical and cost-effectiveness of inpatient mental health rehabilitation services provided by the NHS and independent sector (ACER): Protocol. BMC Psychiatry.

[21] VanMeerten N.J., Harris J.I., Nienow T.M., Hegeman B.M., Sherburne A., Winskowski A.M., Schumacher M., Sponheim S.R. (2013). Inpatient utilization before and after implementation of psychosocial rehabilitation programs: Analysis of cost reductions. Psychol. Serv..

[22] Gonda T., Deane F.P., Murugesan G. (2012). Predicting clinically significant change in an inpatient program for people with severe mental illness. Aust. N. Z. J. Psychiatry.

[23] Sim A., Poremski D., Su A. (2022). Quality Improvement Approach to Increasing Psychiatric Rehabilitation in the Inpatient Setting. Psychiatr. Serv..

[24] Pinkney A.A., Gerber G.J., Lafave H.G. (1991). Quality of life after psychiatric rehabilitation: The clients’ perspective. Acta Psychiatr. Scand..

[25] Awara M.A., Downing L.M., Edem D., Lewis N., Green J.T. (2023). Three-year-cohort-study: Clinical and cost effectiveness of an inpatient psychiatric rehabilitation. Front. Psychiatry.

[26] Awara M.A., Simon P., Lewis N., Edem D., Morrison J.M. (2017). Psychiatric Rehabilitation: Quality of Care and Clinical Effectiveness. J. Psychosoc. Rehabil. Ment. Health.

[27] Parker S., Arnautovska U., Siskind D., Dark F., McKeon G., Korman N., Harris M. (2020). Community-care unit model of residential mental health rehabilitation services in Queensland, Australia: Predicting outcomes of consumers 1-year post discharge. Epidemiol. Psychiatr. Sci..

[28] Koval R.D., Mcdonagh J., Grubaugh A., Young W., Corcoran B., Lee A., Dumas B., Edlund B. (2016). Implementation of Recovery Programming on an Inpatient Acute Psychiatric Unit and Its Impact on Readmission. J. Addict. Nurs..

[29] Paul G.L., Menditto A.A. (1992). Effectiveness of inpatient treatment programs for mentally ill adults in public psychiatric facilities. Appl. Prev. Psychol..

[30] Vanzetto S., Zabotto M., Fasciana F., Varinelli A., Cirnigliaro G., Ferrara L., Dell’oSso B., Viganò C. (2021). Structured Evaluation of Rehabilitation Programs Outcomes in Psychiatry: Application of a Recovery-Centered Model. Psychiatr. Q..

[31] Tsoutsoulis K., Maxwell A., Menon Tarur Padinjareveettil A., Zivkovic F., Rogers J.M. (2020). Impact of inpatient mental health rehabilitation on psychiatric readmissions: A propensity score matched case control study. J. Ment. Health.

[32] Killaspy H., Dalton-Locke C. (2023). The growing evidence for mental health rehabilitation services and directions for future research. Front. Psychiatry.

[33] Edwards M.L., Morris N.P. (2024). How Inpatient Psychiatric Units Can Be Both Safe and Therapeutic. AMA J. Ethics.

[34] Bond G.R., Drake R.E., Becker D.R. (2008). An update on randomized controlled trials of evidence-based supported employment. Psychiatr. Rehabil. J..

[35] Drake R.E., McHugo G.J., Bebout R.R., Becker D.R., Harris M., Bond G.R., Quimby E. (1999). A randomized clinical trial of supported employment for inner-city patients with severe mental disorders. Arch. Gen. Psychiatry.

[36] Sklar M., Groessl E.J., O’Connell M., Davidson L., Aarons G.A. (2013). Instruments for measuring mental health recovery: A systematic review. Clin. Psychol. Rev..

[37] Barlattani T., Mantenuto S., D’amelio C., DI Berardo A., Capelli F., Leonardi V., Socci V., Rossi R., Rossi A., Pacitti F. (2024). Social Cognition and Covid-19: A rapid scoping review. Riv. Psichiatr..

