# Supplementary material for: Components and Effectiveness of Adult Inpatient Psychiatric Rehabilitation Programs: A Scoping Review
Source: Healthcare (Basel). 2025 Nov 19;13(22):2971. doi: 10.3390/healthcare13222971 (PMC12652770; doi:10.3390/healthcare13222971)
Supplement: Supplementary file 1 [file healthcare-13-02971-s001.zip › healthcare-3927245-supplementary.pdf]

Supplementary Materials

Table S1 . Complete search strategies for each database

Database	Search Strategies
Medline (PubMed), n=641	("Psychiatric Rehabilitation"[Mesh] OR "mental health rehabilitation" OR "psychosocial rehabilitation" OR "recovery-oriented care") AND ("Inpatients"[Mesh] OR "hospital-based" OR "inpatient unit*" OR "residential care" OR "psychiatric hospital*") AND ("Adult"[Mesh] OR adults OR "serious mental illness" OR SMI) AND ("Program evaluation" OR "treatment outcome"[Mesh] OR effectiveness OR "service delivery" OR "functional outcome*" OR "community reintegration")
Web Of Science, n=293	TS=("psychiatric rehabilitation" OR "psychosocial rehabilitation" OR "mental health rehabilitation" OR "recovery-oriented care") AND TS=("inpatient*" OR "psychiatric hospital*" OR "residential unit*" OR "hospital-based mental health") AND TS=("adult*" OR "serious mental illness" OR SMI) AND TS=("effectiveness" OR "program evaluation" OR "treatment outcome" OR "functional recovery" OR "readmission")
PsycINFO, n=141	("psychiatric rehabilitation" OR "psychosocial rehabilitation" OR

	"recovery-oriented care" OR "mental health rehabilitation")<br>AND<br>("inpatient" OR "residential treatment" OR "psychiatric hospital" OR "hospital-based care")<br>AND<br>("adult" OR "serious mental illness" OR "SMI")<br>AND<br>("effectiveness" OR "program evaluation" OR "treatment outcome" OR "functional recovery" OR "readmission")
Google Scholar, n=401	"inpatient psychiatric rehabilitation" OR "residential psychiatric treatment" "adult" "effectiveness" OR "readmission" OR "functional outcomes"

Table S2. PRISMA-Scr checklist.

Preferred Reporting Items for Systematic reviews and Meta-Analyses extension for Scoping Reviews (PRISMA-ScR) Checklist

SECTION	ITEM	PRISMA-ScR CHECKLIST ITEM	REPORTED ON PAGE #
**TITLE**			
Title	1	Identify the report as a scoping review.	1
**ABSTRACT**			
Structured summary	2	Provide a structured summary that includes (as applicable): background, objectives, eligibility criteria, sources of evidence, charting methods, results, and conclusions that relate to the review questions and objectives.	1
**INTRODUCTION**			
Rationale	3	Describe the rationale for the review in the context of what is already known. Explain why the review questions/objectives lend themselves to a scoping review approach.	Click here to enter text.
Objectives	4	Provide an explicit statement of the questions and objectives being addressed with reference to their key elements (e.g., population or participants, concepts, and context) or other relevant key elements used to conceptualize the review questions and/or objectives.	Click here to enter text.
**METHODS**			
Protocol and registration	5	Indicate whether a review protocol exists; state if and where it can be accessed (e.g., a Web address); and if available, provide registration information, including the registration number.	Click here to enter text.
Eligibility criteria	6	Specify characteristics of the sources of evidence used as eligibility criteria (e.g., years considered, language, and	Click here to enter text.

SECTION	ITEM	PRISMA-ScR CHECKLIST ITEM	REPORTED ON PAGE #
		publication status), and provide a rationale.	
Information sources*	7	Describe all information sources in the search (e.g., databases with dates of coverage and contact with authors to identify additional sources), as well as the date the most recent search was executed.	Click here to enter text.
Search	8	Present the full electronic search strategy for at least 1 database, including any limits used, such that it could be repeated.	Click here to enter text.
Selection of sources of evidence†	9	State the process for selecting sources of evidence (i.e., screening and eligibility) included in the scoping review.	Click here to enter text.
Data charting process‡	10	Describe the methods of charting data from the included sources of evidence (e.g., calibrated forms or forms that have been tested by the team before their use, and whether data charting was done independently or in duplicate) and any processes for obtaining and confirming data from investigators.	Click here to enter text.
Data items	11	List and define all variables for which data were sought and any assumptions and simplifications made.	Click here to enter text.
Critical appraisal of individual sources of evidence§	12	If done, provide a rationale for conducting a critical appraisal of included sources of evidence; describe the methods used and how this information was used in any data synthesis (if appropriate).	Click here to enter text.
Synthesis of results	13	Describe the methods of handling and summarizing the data that were charted.	Click here to enter text.
**RESULTS**			

SECTION	ITEM	PRISMA-ScR CHECKLIST ITEM	REPORTED ON PAGE #
Selection of sources of evidence	14	Give numbers of sources of evidence screened, assessed for eligibility, and included in the review, with reasons for exclusions at each stage, ideally using a flow diagram.	Click here to enter text.
Characteristics of sources of evidence	15	For each source of evidence, present characteristics for which data were charted and provide the citations.	Click here to enter text.
Critical appraisal within sources of evidence	16	If done, present data on critical appraisal of included sources of evidence (see item 12).	Click here to enter text.
Results of individual sources of evidence	17	For each included source of evidence, present the relevant data that were charted that relate to the review questions and objectives.	Click here to enter text.
Synthesis of results	18	Summarize and/or present the charting results as they relate to the review questions and objectives.	Click here to enter text.
**DISCUSSION**			
Summary of evidence	19	Summarize the main results (including an overview of concepts, themes, and types of evidence available), link to the review questions and objectives, and consider the relevance to key groups.	Click here to enter text.
Limitations	20	Discuss the limitations of the scoping review process.	Click here to enter text.
Conclusions	21	Provide a general interpretation of the results with respect to the review questions and objectives, as well as potential implications and/or next steps.	Click here to enter text.
**FUNDING**			
Funding	22	Describe sources of funding for the included sources of evidence, as well as sources of funding for the scoping review. Describe the role of the funders of the scoping review.	Click here to enter text.

JBI = Joanna Briggs Institute; PRISMA-ScR = Preferred Reporting Items for Systematic reviews and Meta-Analyses extension for Scoping Reviews.
* Where *sources of evidence* (see second footnote) are compiled from, such as bibliographic databases, social media platforms, and Web sites.
† A more inclusive/heterogeneous term used to account for the different types of evidence or data sources (e.g., quantitative and/or qualitative research, expert opinion, and policy documents) that may be eligible in a scoping review as opposed to only studies. This is not to be confused with *information sources* (see first footnote).
‡ The frameworks by Arksey and O'Malley (6) and Levac and colleagues (7) and the JBI guidance (4, 5) refer to the process of data extraction in a scoping review as data charting.
§ The process of systematically examining research evidence to assess its validity, results, and relevance before using it to inform a decision. This term is used for items 12 and 19 instead of "risk of bias" (which is more applicable to systematic reviews of interventions) to include and acknowledge the various sources of evidence that may be used in a scoping review (e.g., quantitative and/or qualitative research, expert opinion, and policy document).

From: Tricco AC, Lillie E, Zarin W, O'Brien KK, Colquhoun H, Levac D, et al. PRISMA Extension for Scoping Reviews (PRISMAScR): Checklist and Explanation. Ann Intern Med. 2018;169:467–473. doi: 10.7326/M18-0850.

Table S3. Details of the identified studies

**Authors**	**Population sample**	**Type of unit**	**Key components of psychiatric rehabilitation program**	**Elements of effectiveness**	**Limitations**	**Main outcomes identified by the authors**	**Quality assessment**

(VanMeerten et al., 2013) [21]	n=311, veterans with serious mental illness	Inpatient services at a VA medical center	New PSR interventions between 2002 and 2008 implemented, including programs in CBSST, WMR, supported employment, and family psychoeduca-tion.	Veterans with SMI who accessed PSR services used less inpatient care and more outpatient care, resulting in an annual cost decrease of $17,739 per hospitalized veteran. PSR interventions led to net savings of $5,378 annually per enrolled veteran, considering both reduced inpatient use and increased outpatient use. Without PSR services, care costs	The study cannot conclusively determine if cost changes were directly caused by PSR interventions or by veterans' individual treatment choices.<br><br>The absence of a randomized group assignment or an active control condition limits the	Analyses showed that providing PSR services to hospitalized veterans with SMI resulted in shorter hospitalizations and cost savings of $17,739 per veteran per year in total mental health care.	Moderate

				increased by $6,325 annually. Thus, enrolling in PSR resulted in a total annual savings of $11,703 per hospitalized veteran. At the medical center, PSR programs led to total cost reductions of $545,259 per year, driven by decreases in inpatient and PPH costs and increases in outpatient costs.	ability to conclude that cost reductions wouldn't occur with non-PSR interventions.<br><br>There may be unmeasured factors influencing reduced inpatient usage by veterans, such as additional treatments (e.g., medication management, individual therapy,		

					etc.) that were available to all veterans, not just those enrolled in PSR.<br><br>While MHICM was considered, its impact was ruled out, as it did not significantl y affect hospitalizat ions during the study period.<br><br>The sample		

					is predominantly Caucasian and male, limiting generalizability to more diverse or non-veteran populations.<br><br>The study did not explore how cultural factors might affect the response to PSR or how continuity of care		

					could be maintained outside the VA system.<br><br>Veterans in the PSR group used a combination of interventions, making it difficult to attribute findings to any one specific intervention.<br><br>The study's findings are specific to the VA Medical		

					Center studied, limiting broader applicabilit y.<br><br>The study did not examine the long-term effects on disability reduction or overall illness progression , which may mean that cost reductions were underrepre sented.<br><br>The study did not		

					provide a breakdown of costs for each PSR program, making it difficult to compare the cost-efficiency of individual interventions.<br><br>Future research should focus on examining how different interventions in PSR affect effectiveness and cost reduction.		

(Killaspy et al., 2024) [20]	n= 500, patients of inpatient units in both the NHS and independent sector	inpatient mental health rehabilitation units in the NHS and independent sectors across England	investigate the clinical and cost-effectiveness of inpatient mental health rehabilita-tion provided by the NHS and independent sector with the objective of investigating differences between them in terms of: patient characteristics; service quality; patient, carer and staff experiences; clinical	This study is going until 2026. No data yet available. Article simply reports about how the data will be analyzed and the objectives of the study.	Selection bias, participants were paid 20 pounds to participate.		High

			effectiveness; and their relative cost effectiveness.				

(Pinkney et al., 1991) [24]	n= 55, patients discharged from inpatient psychiatric rehabilitation programs	Inpatient psychiatric programs from Brockville Psychiatric Hospital	Uses CQLI to evaluate patients lives 1-year post hospitalization in inpatient unit. 2 rehab programs from Brockville Psychiatric Hospital. 1 being an adaptation of Fairweather psychiatric rehab model. The other being based on a rehab model developed at Boston Universoty. Both psychiatric	96% of patients saw their quality of life improve as a consequence of leaving the hospital. Results summarized (exact % in results section): People discharged from psychiatric rehabilitation programs reported improved quality of life, adjusting well to community living. They worked part-time, attended	Although not explicitly stated: small sample size. Study taken from 1990. Patients taken from specific hospital in Ontario: should account for social programs available in said province and Canada (as a whole) (social programs in Canada allowing	Proper integration into community setting post-hospitalisation. Most people had the basic skills needed to live in the community. They behaved well and easily fit into the larger community. They also used support services from the hospital to help them adjust to community life.	Low

			programs tought community living skills, focused on coping skills, not symptom reduction. Programs also offered outpatient services for those requiring support.	school, participated in leisure activities, and had adequate housing, with most remaining out of the hospital. Despite challenges like poverty, stigma, and securing employment, they benefited from ongoing support from treatment teams. Deinstitutionalization did not lead to homelessness or criminal behavior. While many	for quicker adjustment, public healthcare ++ (therefore possibly having a greater willingness to use outpatient services)		

				were readmitted briefly to hospitals due to acute symptoms, this was not a failure of the system. Participants needed more help with interpersonal skills and employment, as vocational rehab programs were not effective.			

(Parker et al., 2020)[27]	n= 501, consumers admitted to five CCUs in Queensland, Australia between 2005 and 2014.	Patients of five community care units (CCU)	Modern CCUs offer short-term, clinically-focused residential psychiatric support aimed at improving various areas of personal functioning, mainly living skills and community integration, within the broader context of mental health (other quotes: Parker et al., 2019). These grouped,	first quantitative evaluation of community-based mental rehabilitation services in Australia. It found that 43% of consumers showed improvements in mental health and social functioning after receiving CCU care. Consumers with worse mental health and longer care durations were more likely to show progress.	Use of retrospective data which imited variable analysis. Incomplete adherence to routine outcomes monitoring protocols. Outcome measures like HoNOS may not fully reflect clinically significant impairments in specific domains. "Regression-to-the-mean" and external	Group-level analyses revealed significant improvements in mental health, social functioning, and reductions in psychiatry-related bed-days, ED presentations, and involuntary treatment. However, there were no significant changes in disability or accommodation instability. Additionally, 54.7% of	Moderate

			independent living units provide 24-hour rehabilitation assistance to individuals whose daily functioning is impacted by severe mental illness (author quotes: Meehan et al., 2017)	Significant improvements were seen in mental health, social functioning, hospital bed days, and ED visits, but no improvement was found in accommodation instability or disability. While the reduction in HoNOS scores was consistent with previous studies, the lack of improvement in disability indicates that CCU care may be more effective in	factors should be considered when interpreting findings. The predictive models explained only part of the variability in consumer improvement. Medication adherence and formal education were not included in the analysis due to missing data. The study	consumers showed reliable improvement in mental health and social functioning, while 43.0% demonstrated RCS improvement.	

				clinical recovery than functional recovery. The study also highlighted that some consumers experienced deterioration despite rehabilitation, and factors like discharge stress and access to services may contribute. Regression analysis identified that longer CCU stays and higher baseline severity were associated with greater	lacked detailed information on specific therapeutic approaches used in rehabilitation. More research is needed to identify active therapeutic processes that lead to positive outcomes.		

				improvement, while pre-admission disability and bed use were linked to less progress. Overall, the study suggests that while CCU care leads to positive outcomes for many, challenges remain in improving long-term functional recovery, especially for those with schizophrenia. Future research should focus on individual			

				change and explore factors contributing to deterioration despite rehabilitation.			

(Awara et al., 2023)[25]	n=158, patients who had been discharged within 3 years and 1 month period between 1 June 2012 and 30 June 2015 in NS, Canada.	inpatient rehabilitation service in NS, Canada	Whole-system approach of the biopsychosocial rehabilitation model. More details unspecified	The service utilization of acute psychiatric services, including admission rates, length of stay, and ER visits, for each patient was examined over a 1-year period, comparing pre- and post-exposure to rehabilitation treatment in the unit in a bi-directional manner.	Small sample size. The high prevalence of other psychiatric and medical co-morbidities in the studied cohort means that the sample's heterogeneity could influence the outcome. Non-randomized nature f study and no control group.	During the study period, 185 patients were admitted, with 158 being discharged. A significant reduction was observed in readmission rates (a 64% decrease), length of stay (6,585 fewer hospital days), and emergency room visits (166 fewer visits) ($P < 0.0001$). These improvemen	High

						ts led to notable cost savings in the year following rehabilitation. The substantial decrease in readmission rates, hospital stay duration, and ER visits can be considered cost-effective, leading to a savings of CAD 9,270,000 in the year following rehabilitation.	

(Killaspy and Dalton-Locke, 2023) [32]	n = 147, National Psychosis Unit	National Psychosis Unit	Similar to an inpatient rehabilitation service in delivering specialized treatment for individuals with complex psychosis; however, it functions at a national level rather than within a local NHS Trust. (sample taken from UK)	average number of inpatient days decreased from 335 days (SD 273) before admission to the National Psychosis Unit to 199 days (SD 262) afterwards.	Small sample size. Short before and after periods. No control group, therefore "regression to mean" might explain results.	average number of inpatient days decreased from 335 days (SD 273) before admission to the National Psychosis Unit to 199 days (SD 262) afterwards.	Moderate

(Gonda et al., 2012) [22]	n = 337, 170 patients admitted to the male unit and 167 patients admitted to the female unit over the study period	inpatient units at Bloomfield Hospital, Orange, New South Wales, Australia. Assessed at admission, 3-month reviews and discharge using the expanded Brief Psychiatric Rating Scale, the Health of the Nation Outcome Scales and the Kessler 10.	Team made up of a psychiatrist, psychologist, social worker, diversional therapists, occupational therapist, clinical nurse consultant, a unit manager, and nursing staff for each ward. Patients encouraged to take part in group sessions that matched their needs (topics like drug and alcohol use,	Psychiatric Symptomatology (BPRS-E):<br><br>Reliable Improvement: 84 patients (48.6%) showed reliable improvement in psychiatric symptoms. No Reliable Deterioration: No patients experience reliable deterioration<br><br>Clinically Significant Improvement (RCS): Using a cutoff value of 59, 62 patients (35.8%)	Research was conducted at one location, limiting the generalizability of the findings to other settings or populations. Brief follow-up period. Specific scales (BPRS-E, HoNOS, K10), which may not fully capture all aspects of mental health or recovery. potential	32.4% of inpatients showed significant improvement in psychiatric symptoms, 19.5% in psychosocial functioning, and 20.2% in psychological distress. Logistic regression identified predictors for improvement in psychiatric symptoms, but not in psychosocial functioning or distress. Patients with	Moderate

			medication education, and anger management). Pts had individualized care plans, and discharges were generally planned in advance	showed RCS improvement. Using a cutoff value of 52, 56 patients (32.4%) showed RCS improvement.<br><br>Conducting Logistic Regression: For the improvement group, the model accurately classified 62.9% of the cases while for the no improvement group, the model correctly classified 74.6% of the cases. The	factors that could influence patient outcomes, such as the severity of illness or treatment adherence not accounted for. other relevant factors, such as socio-economic status or treatment environment, were not explored.	schizoaffective disorder had better improvement in psychiatric symptoms than those with schizophrenia. Co-occurring substance abuse was linked to greater improvement. cf 'Key components' column for more details. But these were main identified outcomes	

				model also classified 69% of the cases.<br><br>Primary Diagnosis: Compared to schizophrenia patients, those with schizoaffective disorder were 3.5 times more likely to improve.<br><br>Co-morbid Diagnosis: Substance abuse disorders were associated with higher probability of improvement, but this finding is not			

				statistically significant.<br><br>Psychosocial Functioning (HoNOS): Reliable Improvement: 100 patients (30.4%) showed reliable improvement in psychosocial functioning and 3 patients (0.9%) showed reliable deterioration. Statistically significant: The model was not statistically significant and			

				accounted for only 4.9% of the variance.<br><br>Clinically Significant Change: Using a cutoff score of 6, 75 patients (22.8%) showed RCS improvement. Using a cutoff score of 5, 64 patients (19.5%) showed RCS improvement.<br><br>Psychological Distress (K10): Reliable Improvement: 83 patients with reliable improvement.			

				RCS was set at 7 for 85 patients. With score threshold of 10, only 41 passed it.			

(Tsoutsoulis et al., 2018) [31]	n = 252, consumers of a nonacute inpatient mental health rehabilitation unit of a metropolitan tertiary care hospital in Sydney, Australia.	inpatient mental health rehabilitation unit	Inpatient unit provides long-term care for people with severe psychiatric conditions, both voluntary and involuntary admissions. Team including psychiatrists, therapists, and nurses, create personalized care plans based on each person's needs and goals. The programs	cf main outcomes for numerical values associated to each outcome. 1. Occurrence of psychiatric readmission within 12 months of discharge: The proportion of consumers experiencing a readmission significantly decreased following inpatient care, aligning with the normative group. 2. Total number of psychiatric readmissions	1. Limitation in the period of time in which this study took place. Hard to tell long-term effects of care. 2. The clinical group was not monitored after discharge, thereby factors like having a job, stable housing, good social support, or quality outpatient care may	These are same outcomes as the 'Elements of effectiveness' column, but with the numerical values given by the authors. (these are the three main outcomes identified in methods section: 1. Occurrence of Psychiatric Readmission within 12 Months of Discharge: Before receiving	Moderate

			focus on daily life skills, social skills, work, health, and family support. Consumers also join group programs that cover self-care, symptom management, substance use, and personal growth.	within 12 months of discharge: The number of readmissions also significantly decreased, approaching normative group levels, except in consumers with comorbid bipolar disorder, substance use disorder, or personality disorder.<br>3. Time to psychiatric readmission after discharge: The time to a	have affected readmission.<br>3. variability between episodes of care.<br>4. Results from only one unit of care.	inpatient rehabilitation, all consumers (100%) had experienced at least one psychiatric readmission in the previous 12 months. Following discharge, this figure dropped significantly to 33%, indicating a substantial improvement ($\chi^2(1) = 250.53$, $p < 0.01$).<br><br>2. Total Number of Psychiatric	

				readmission significantly increased following inpatient care, approximating normative group values, and was related to the number of previous admissions.		Readmissions within 12 Months of Discharge: The average number of readmissions per consumer declined from 1.48 before rehabilitation to 0.58 after discharge. This reduction was statistically significant (t(251) = 11.07, p < 0.01), highlighting the positive impact of the inpatient	

						program.<br><br>3. Time to Psychiatric Readmission after Discharge: The average time until readmission increased from 110 days before inpatient care to 152 days afterward—a meaningful and statistically significant extension ($t(83) = 2.78$, $p = 0.01$). These were the three outcomes	

						identified as per the authors	

(Koval et al., 2016)[28]	n= 191 admissins between July-September 2013	16-bed inpatient adult mental health unit in a Veterans Affairs Medical Center	Slightly unclear, however: 16-bed inpatient locked unit. Recovery implementation plan best suited for success (uncertain as to what this means)	Decrease in readmission percentages was seen with implementation of recovery-oriented care when comparing the same 3-month period over 3 years	1. Quality improvement project more than a research study, therefore confounding variables were possible<br>2. Staff turnover, nonrecovery training initiatives put into place, which could have influenced data over the 3 year period.<br>3. Engagement and actual hours spent	Between July–September 2013, there were 191 admissions, with 25 readmissions (13.1%). In 2014, during the same period, 166 admissions occurred, and 15 were readmissions (9.0%), showing a 31.3% decrease in readmission rates after recovery-oriented care was introduced. By July–September	Moderate

					by veterans in in-unit services were not precisely accounted for, possibly preventing the veterans from achieving optimal care.<br>4. Training of staff was completed *after* this recovery-care oriented care was implemented<br>5. Specific patient information	2015, there were 136 admissions, with 10 readmissions (7.4%), marking an overall 43.5% decrease in readmissions from 2013. However, August 2015 had an outlier, with 7 readmissions out of 40 admissions (17.5%), the highest readmission percentage during the study.	

					was not gathered. Various factors not accounted for and no risk adjustments made.		

(Edwars and Morris, 2024) [33]	Moreso an opinion article. Not a particular research sample size.	Opinion article. Looks at evolution of inaptient units. Frequency of adverse events in said units, safety funnel, equity, future directions	N/A	N/A	N/A	Frequency of adverse events in inpatient psychiatric units are reported. Adverse events in inpatient psychiatric settings, including self-harm, violence, and privacy breaches, are common. International data shows a suicide rate of about 147 per 100,000 psychiatric inpatient years, with suicide rates	Moderate

						for inpatients nearly 50 times higher than the general population. Between 4% to 70% of patients engage in nonsuicidal self-injury. A review found that 17% of patients commit physical violence in inpatient settings, while 25% to 85% of staff report experiencing physical aggression.	

						These events can lead to legal repercussions, including sanctions and lawsuits, with failure to protect patients from self-harm being a common legal issue.<br><br>Safety funnel paragraph: The "Safety Funnel" concept describes how psychiatric units focus more on safety than therapeutic	

						needs due to pressure from the risk of adverse events and legal consequences. To prevent incidents like suicides with shoelaces or intoxication from hand sanitizer, units often remove or restrict certain items. While these measures are meant to keep patients safe, they can create environment	

						s that are less supportive of recovery. For example, removing privacy curtains or exercise equipment may affect patients' privacy and well-being. Additionally, safety features like locked doors and seclusion rooms can cause fear and distress among patients, making it hard to balance	

						safety with healing. (authors mention how pts sometimes refer to themselves as 'survivors' of said units due to distress caused by aforementio nned measures)	
(Bunyan et al., 2016) [19]	n = 24 individuals discharged from the three units between 1 October 2009 and 30 September 2010.	three in-patient rehabilitatio n units across one London National Health Service trust	N/A	There was a significant reduction in hospital admission days during the two years following rehabilitation compared to the two years before,	1. Lack of control group<br>2. No randomised control trial for determinin g causality.<br>3. Although linear regression	Patients had a statistically significant reduction in hospital admission days in the 2 years after rehabilitatio n compared to the 2 years before	Moderate

				equally leading to a noticeable decrease in bed costs. A longer duration of rehabilitation placement was linked to fewer admission days afterward. A large portion of the sample transitioned to more independent living, with some individuals having no further admissions at follow-up.	analysis did not identify age or gender as significant predictors of hospital bed days or associated costs, the limited sample size restricted any meaningful evaluation of clinical or demographic predictors of outcome (small sample size essentially)	(t(21) = 3.052, P = 0.006).<br><br>Hospital admission costs were also significantly lower post-rehabilitation.<br><br>Five patients who had been continuously admitted before rehabilitation stayed longer in rehab (average 953 vs. 701 days); 4 were discharged to residential	

						care, 1 to an independent flat, and 3 had no readmissions in follow-up.<br><br>Another 5 patients required 3–4 years of rehabilitation and were discharged to residential care; none were readmitted in the 2-year follow-up.<br><br>Of the 11 patients who stayed less than 18 months, 2 were	

						discharged to long-term inpatient care; these were the only patients who entered continuous inpatient care after rehab, possibly indicating early recognition of limited rehab benefit.	

(Vanzetto et al., 2021) [30]	n = 111 patients from a University High Assistance Rehabilitation Community (C.R.A.) based in Milan	Inpatient unit.	24 /7 healthcare assistance to patients with major psychiatric disorders such as psychotic disorders, afective disorders and severe personality disorders. Includes two-programs: 1) Post-Acute (RPA): A 3-month program, renewable up to 6 months, designed for patients recovering	The CRA rehabilitation program significantly improved global functioning, with reduced hospitalizations, better continuity of care, stable adherence to LAI antipsychotics, and maintained employment during the year after discharge. (CRA: University High Assistance Rehabilitation Community)	1. Small sample size. One public Italian Community causing undererpresentation of psychiatric patients. 2. Use of psychiatric patients with mixed dx at different stages of disease, thereby possibly confounding outcomes. 3. Authors suggest that there should be a creation of	This study confrmed the utility of a structured outcome indicator model and highlighted its feasibility in daily clinical context of a rehabilitative community. 1. Before admission, the average number of hospitalizations was 1.8±1.5 (min=0, max=8), which decreased to 0.4±0.8 (min=0,	High

			from an acute psychiatric episode.<br>2) High Intensity (RHI): An 18-month program, extendable to 24 months, aimed at individuals requiring a more structured and specific rehabilitative pathway.		standardized outcomes model in diferent settings.	max=4) after discharge. In the RHI group, hospitalizations decreased from 1.6±1.5 before to 0.4±0.9 after, while in the RPA group, the number went from 2.4±1.5 before to 0.4±0.8 at discharge. The reduction was statistically significant ($p < 0.001$) in all subgroups.<br>2. The average duration of	

						hospitalization per year in the total sample was 35.8±34.3 days before admission (min=0, max=193), compared to 7±17.3 days per year at the end of the program (min=0, max=100). In the RHI group, the average duration was 29.2±29.4 days before admission and 6.1±13.2 days in the following 12 months. In	

						the RPA group, the mean duration was 52.5±40.1 days before, compared to 9.1±24.4 days after the rehabilitation program. The reduction was statistically very significant ($p < 0.001$) in all subgroups. 3. Continuity of care is defined as at least one psychiatric examination every 90 days in a 12-	

						month period. In the overall sample, 53.2% of patients received regular outpatient care in the year before admission, while 95.5% maintained regular contact with CPS caregivers after discharge, showing a significant increase (p < 0.001). In the RHI group, continuity of care improved	

						from 43.6% before admission to 97.4% after discharge ($p < 0.001$). In the RPA group, continuity of care increased from 75.6% to 90%, though this change was not statistically significant.	

(Awara et al., 2017)[26]	n = 80 patients	Study conducted in a 40-bed-inpatient rehabilitation unit retrospectively over a 1-year period.	Unit staffed with inter-discplinery team: the inpatient program included social workers, occupational therapists, occupational therapy assistants, recreation/arts therapists, an attending psychiatrist, psychiatric residents, a medical doctor (Family Physician), care team assistants, and a health promotion	The study found that the integrated, multidisciplinary rehabilitation approach led to a reduction in length of stay (LOS) and bed occupancy. It also decreased the frequency of emergency room visits, readmissions, and reduced LOS in acute services.	1. Small sample size<br>2. Short pre and post exposure to tx might have been too short (6 months) to draw proper conclusions.	1. Medication change: 51% (41 patients); 27 discharged with 136-day mean LOS (79 fewer days) vs. 31 patients with no medication change (mean LOS 215 days). cf figure 1. good visual rep.<br>2. LOS comparison (admission before vs. during study): Pre-study: 335 ± 263 days	Moderate

			coordinator. Specific program services not listed. Bio-psychosocial mdoel was used.			Post-study: 78 ± 61 days (257-day reduction, p = 0.0001).<br>3. Bed occupancy: Reduced from 39 to 25 beds (26% reduction) during the study period Further reduced to 15 beds (62% reduction) in the 6 months after.<br>4. Acute MH unit admissions: Significant decrease in admissions post-rehab (81% had zero	

						admissions post-rehab). Reduced LOS on acute mental health units post-rehab ($p < 0.0001$). 5. Emergency Room visits: Significant reduction in ER visits post-rehab ($p < 0.005$). 6. Quality of care (QuIRC tool) (strengths of the Unit): Strengths: Living Environment (+11%), Treatments and Intervention	

						s (+10%), Social Interface (+25%). Areas for improvemen t: Self-management and Autonomy (-4%), Human Rights (-5%), Recovery-based Practice (-5%).	
(Sim et al., 2022) [23]	Total number unspecified. 44-bed men’s ward at the Institute of Mental	44-bed inpatient men's unit.	N/A	Prior to the project, referral rates remained consistently low. After implementing the	1. Small sample size 2. Specific population 3. Context: study took place during	Weekly ratio of people successfully referred to inpatient rehabilitatio n services over the	Moderate

	Health, Singapore. The average weekly occupancy was 38±5 patients, with approximately 26±3 patients eligible for referral to rehabilitation services.			interventions, however, referrals increased gradually and significantly, with an average of one additional successful referral every six weeks. This upward trend was maintained throughout the observation period.	COVID-19 (important external factor to consider)<br>4. No control group<br>5. Short term evaluation, long term results not yet looked at<br>6. patient motivations, systemic barriers. Examples of factors not considered.	total number eligible.	

(Paul and Menditto, 1992) [29]	n = 84, divided into three groups (28 each) and assigned to:<br><br>Social-learning program<br>Milieu/therapeutic-community program<br>Traditional hospital treatment	Inpatient psychiatric unit	1. Social-learning program<br>Based on behavioral principles (operant and associative learning).<br>Used token economies, step-levels, and highly structured environments.<br>Emphasized functional skills training, individualized goals, and weaning from reinforcement systems.<br>Minimized psychotropic	1) Social-learning program: Produced the highest rates of sustained improvement in all areas of patient functioning (behavioral, emotional, social).<br>More than 97% of patients achieved successful release (90+ days in the community without rehospitalization).<br>Demonstrated that even patients with long-term	1. Small population size<br>2. Results not necessarily generalizable due to specific population<br>3. Taken from same region<br>4. Not much f-u data<br>5. Article published in 1992. Artcile is also 23 pages long. FIrst page has an editor's note mentionning how this	Social-learning program offered best results.	Moderate

			drug use. Showed most effective and cost-efficient results.<br><br>2.Milieu/therapeutic-community program Emphasized group decision-making, patient responsibility, and peer support. Included daily community meetings and smaller peer groups. Moderately structured; relied more	institutionalization (average of 17 years) could improve substantially and maintain community tenure. Reduced reliance on psychotropic medications. Proven to be cost-efficient by shortening average inpatient stays while increasing the number of patients treated.<br><br>2. Milieu/therapeutic-community	article exceeds (by a lot) the page limit usually given to articles (potential internal bias)		

			on social norms and expectations. Variable use of psychotropic drugs. Less effective than social-learning, but better than traditional treatment.<br><br>3. Individualized supportive care (traditional hospital tx) characterized by emphasis on individual and/or group	program: Showed moderate effectiveness, better than traditional treatment but not as effective as the social-learning approach. Improvement was inconsistent, and only about 55% of patients achieved sustained positive outcomes. Some gains appeared to result from the use of behavioral reinforcement			

			modalities in the absence of a systematic, overriding set psychosocial procedures ad-<br>dressed to all patients.	elements borrowed from social-learning rather than from the core principles of the milieu approach itself.<br><br>3. Traditional hospital treatment: Was the least effective. Only about 45% of patients achieved significant post-discharge success. Treatment outcomes were inconsistent,			

				and improvements were not clearly linked to any specific therapeutic procedures. Heavy reliance on medications with limited psychosocial intervention and little structure or skill-building.			
